# Potential Antimetastatic Effect of Timosaponin AIII against Human Osteosarcoma Cells through Regulating the Integrin/FAK/Cofilin Axis

**DOI:** 10.3390/ph14030260

**Published:** 2021-03-14

**Authors:** Yi-Hsien Hsieh, Wen-Hung Hsu, Shun-Fa Yang, Chung-Jung Liu, Ko-Hsiu Lu, Pei-Han Wang, Renn-Chia Lin

**Affiliations:** 1 Institute of Medicine, Chung Shan Medical University, Taichung 40201, Taiwan; hyhsien@csmu.edu.tw (Y.-H.H.); ysf@csmu.edu.tw (S.-F.Y.); u9807410@gmail.com (P.-H.W.); 2 Department of Medical Research, Chung Shan Medical University Hospital, Taichung 40201, Taiwan; 3 Division of Gastroenterology, Department of Internal Medicine, Kaoshiung Medical University Hospital, Kaoshiung, Medical University, Kaoshiung 80756, Taiwan; s3392388@ms15.hinet.net (W.-H.H.); 1020590@ms.kmuh.org.tw (C.-J.L.); 4 Department of Orthopedics, Chung Shan Medical University Hospital, Chung Shan Medical University, Taichung 40201, Taiwan; cshy307@csh.org.tw; 5 School of Medicine, Chung Shan Medical University, Taichung 40201, Taiwan

**Keywords:** TSAIII, human osteosarcoma cell lines, cofilin, metastasis, F-actin

## Abstract

Timosaponin AIII (TSAIII) is a steroidal saponin which demonstrates anti-tumour activities. However, the effect of TSAIII on human osteosarcoma cells remains largely unknown. In this study, we demonstrated that TSAIII exerted a significant inhibitory effect on the distribution of cytoskeletal F-actin and cytoskeletal-related proteins, which contributed to the suppression of cell migration and invasion, without inhibiting cell growth or apoptosis. In the synergistic inhibitory analysis, cotreatment of TSAIII with αVβ3 integrin inhibitor [Cyclo(RGDyK)] or focal adhesion kinase (FAK) inhibitor (PF-573228) exerted greater synergistic inhibitory effects on the expression of Intergin αVβ3/FAK/cofilin axis, thus inhibiting the migration and invasion capacities of human osteosarcoma cells. TSAIII was demonstrated to significantly inhibit the pulmonary metastasis formation of human osteosarcoma cells in vivo in metastasis animal models. These findings reveal the inhibitory effects of TSAIII on the metastasis progression of human osteosarcoma cells and the regulation of integrin-αVβ3-FAK-Src and TESK1/p-cofilin mediated cytoskeletal F-actin pathway. Therefore, TSAIII might represent a novel strategy for the auxiliary treatment of human osteosarcoma cells.

## 1. Introduction

Osteosarcoma is the most common malignant bone tumour that primarily occurs in childhood and adolescence [1]. Surgical resection is the typical treatment modality for osteosarcoma. Adjuvant chemotherapy has been demonstrated to greatly improve the prognosis for patients with osteosarcoma by increasing the five-year survival rate from 20% to approximately 55–70% [2]. However, the five-year survival rate remains dismal at less than 20% in patients with osteosarcoma and metastatic lesions [3]. Therefore, the development of novel treatment strategies that target and prevent metastasis is urgently required to promote patient survival.

Pulmonary metastasis is the most common cause of osteosarcoma, accounting for the poor prognoses and high mortality rates. Metastasis is a complex process that involves several events collectively termed as the invasion–metastasis cascade [4]. Integrins, composed of α and β subunits, contribute to the activation of diverse intracellular signalling molecules, which in turn contribute to the reorganisation of actin filaments and cell locomotion [5,6,7]. Metastasis involves the interaction between cellular integrins and the extracellular matrix (ECM), which leads to the recruitment of signalling molecules and actin filaments to integrins followed by the activation of the integrin-mediated intracellular signal pathway [8]. Proteins such as RhoA, Rac1, and CDC42 belonging to the focal adhesion kinase (FAK), Src family kinase, Erk, Akt/protein kinase B (PKB), and Rho GTPase families are activated by integrin-mediated cell motility [9,10]. The resulting reorganisation of actin filaments regulates downstream effector molecules, including LIM domain kinase (LIMK), cofilin, and testis associated actin remodelling kinase 1 (TESK1), which affect actin polymerisation and intracellular contractility [11,12]. Cofilin plays a critical role in cancer invasion and metastasis [13]. It is a small actin-binding protein regulated by phosphorylation at serine 3. When phosphorylated at serine 3, cofilin is deactivated and cannot bind to actin molecules, thus impairing cytoskeleton remodelling. Cofilin Ser3 is activated through dephosphorylation by slingshot protein phosphatase (SSH) and pyridoxal phosphatase (PDXP) [14,15,16].

Many studies have investigated the potential efficacy of natural phytochemicals against cancers. *Anemarrhena asphodeloides* has been used as a traditional medicine to treat diabetes and haemoptysis in Asian countries. Steroidal saponins are major compounds of *A. asphodeloides*. Spirostanol saponins, which contain a sugar chain at the C3 position, have been reported to exhibit biological activities of anti-inflammation, antiproliferation, antimetastasis, antiangiogenesis, and antimultidrug resistance in vitro and in vivo [17,18,19]. Timosaponin AIII (TSAIII) is a steroidal saponin and has been reported to demonstrate proapoptotic and antimetastatic activities against melanoma, colorectal carcinoma, breast cancer, and nonsmall-cell lung cancer in vitro [20,21,22,23]. However, the effect of TSAIII on cell proliferation and metastasis in osteosarcoma and the underlying molecular mechanisms remain unknown. In this study, we demonstrated that TSAIII inhibits cell migration and invasion through the downregulation of integrin-αvβ3/FAK/Src and induction of phosphorylated cofilin dependent on the F-actin cytoskeleton in human osteosarcoma cells.

## 2. Results

### 2.1. Effect of TSAIII on Cell Viability in Human Osteosarcoma Cells

**Figure 1:**
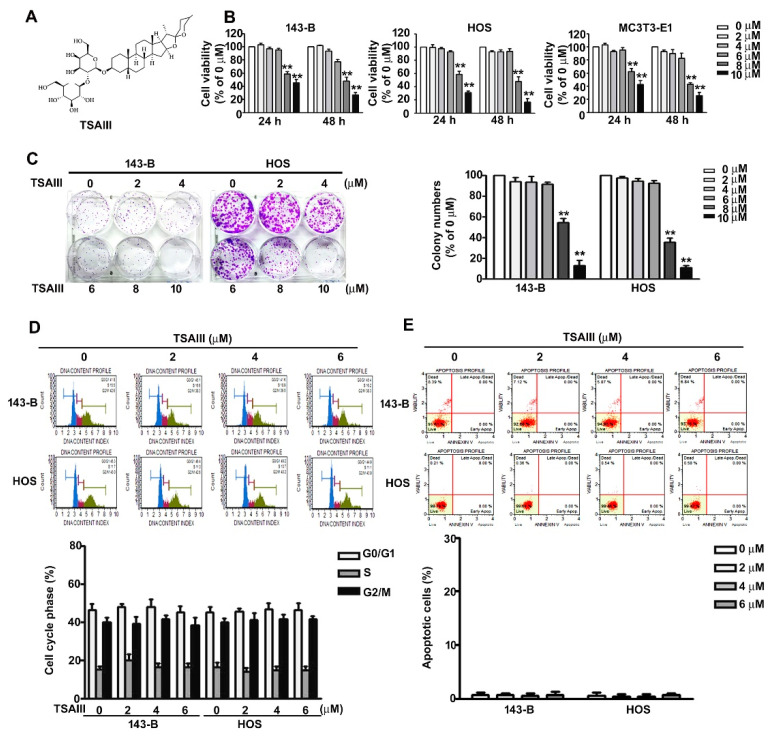
Effect of Timosaponin AIII (TSAIII) on viability and cytotoxicity of human osteosarcoma and normal osteoblast cells. (**A**) Structure of Timosaponin AIII (TSAIII). (**B**) 143-B and HOS cells (human osteosarcoma cells) and MC3T3-E1 cells (normal osteoblasts) were exposed to various concentrations (0, 2, 4, 6, 8, and 10 μM) of TSAIII for 24 h, and cell viability was measured using the CCK8 assay. (**C**) Cell proliferation rate of human 143-B and HOS osteosarcoma cells exposed to TSAIII (0, 2, 4, 6, 8, and 10 μM) for 24 h was measured using a colony formation assay. (**D**) Regulation of cell cycle distribution and (**E**) cell death in human 143-B and HOS osteosarcoma cells exposed to various concentrations (0, 2, 4, and 6 μM) of TSAIII were analysed using Propidium Iodide (PI) staining or Annexin V/PI staining through flow cytometry. ** *p* < 0.01 versus control.

The structure of timosaponin AIII (TSAIII) is presented as in Figure 1A. The effect of TSAIII on cell viability and colony formation was analysed in human 143-B and HOS osteosarcoma cells. Both 143-B and HOS cell lines were treated with various concentrations (0, 2, 4, 6, 8, and 10 μM) of TSAIII for 24 and 48 h and then analysed using the CCK8 assay. Cell viability was significantly reduced by TSAIII (8 or 10 μM) in 143-B cells with an IC50 of 8.6 ± 1.2 μM and 7.8 ± 1.7 μM at 24 and 48 h; HOS cells with an IC50 of 8.3 ± 1.5 μM and 7.2 ± 1.1 μM at 24 and 48 h, respectively. We further measured no significant cell viability in normal osteoblast cell line MC3T3-E1 up to a concentration of 6 µM (Figure 1B). Colony formation was assessed to confirm the inhibitory effect on the growth of 143-B and HOS cells treated with TSAIII (0, 2, 4, 6, 8, and 10 μM) for five days (Figure 1C). These results revealed that high concentrations of TSAIII (8 and 10 μM) exerted a cytotoxic effect on the growth of human osteosarcoma and normal osteoblast cells. Therefore, we used TSAIII at concentrations less than 8 μM in subsequent experiments.

To determine the effect of TSAIII on cell cycle distribution and apoptosis in human osteosarcoma cells, 143-B and HOS cells were treated with various concentrations (0, 2, 4, and 6 μM) of TSAIII for 24 h, and flow cytometry analysis was conducted. The results revealed that TSAIII treatment (2, 4, and 6 μM) had no effect on cell arrest at any phase (Figure 1D). Moreover, the results of the flow cytometry analysis indicated no induction of apoptosis in the 143-B or HOS cells (Figure 1E). Based on these results, TSAIII has no effect on the induction of cell cycle arrest or apoptosis in human osteosarcoma cells.

### 2.2. TSAIII Inhibits Cell Migration, Invasion, and F-Actin Expression in Human Osteosarcoma Cells

**Figure 2:**
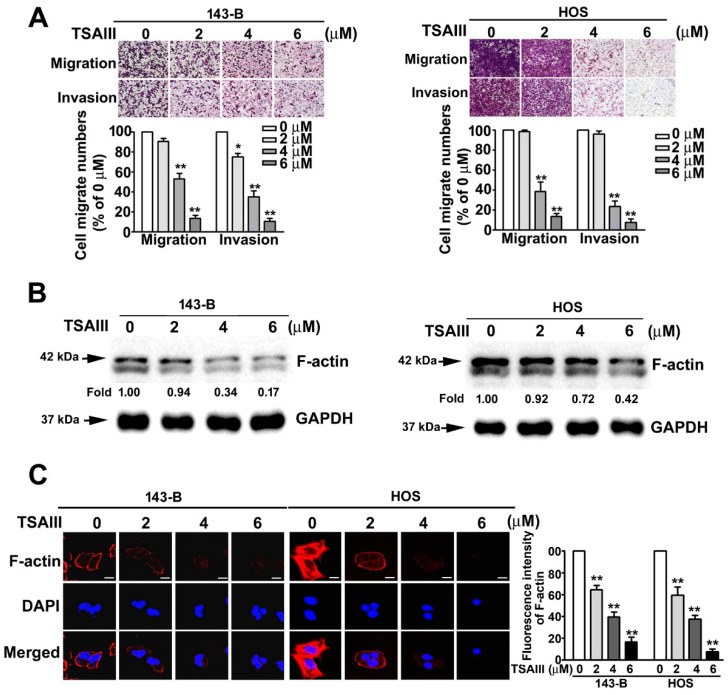
Effect of TSAIII on cell migration, invasion, and F-actin expression of human osteosarcoma cells. (**A**) Human 143-B and HOS osteosarcoma cells were treated with various concentrations of timosaponin AIII (TSAIII; 0, 2, 4, and 6 μM) for 24 h, and cell migration and invasion abilities were measured. (**B**) Cytoskeletal F-actin expression in human 143-B and HOS osteosarcoma cells exposed to TSAIII (0, 2, 4, 6 μM) was measured through immunoblotting. Glyceraldehyde-3-phosphate dehydrogenase (GAPDH) was used as the internal control. (**C**) Distribution of cytoskeletal F-actin in 143-B and HOS cells was further confirmed using immunofluorescence analysis. * *p* < 0.05; ** *p* < 0.01 versus control. Scale bar: 50 μm.

To identify the effect of TSAIII on cell migration and invasion activity in human osteosarcoma cells, we conducted assays after treating 143-B and HOS cells with various concentrations of TSAIII (0, 2, 4, and 6 μM) for 24 h. The results showed that TSAIII significantly suppressed the cell migration and invasion of both human 143-B and HOS cells in a dose-dependent manner (Figure 2A). Cytoskeletal F-actin is crucial for cancer cell migration and invasion. To investigate the effect of TSAIII on F-actin expression in human osteosarcoma cells, both human 143-B and HOS cell lines were treated with various concentrations (0, 2, 4, and 6 μM) of TSAIII for 24 h and analysed through immunoblotting (Figure 2B). The distribution of F-actin in the cell lines was further observed through immunofluorescence analysis (Figure 2C). The results indicated that the expression and distribution of F-actin in the 143-B and HOS cells were significantly reduced in a dose-dependent manner.

### 2.3. TSAIII Suppresses the Expression and Activation of Integrin-Mediated Cytoskeletal-Related Proteins in Human Osteosarcoma Cells

**Figure 3:**
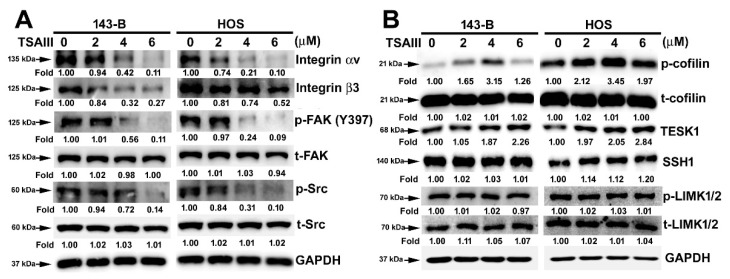
Regulation of TSAIII on integrin-αv, integrin-β3, and cytoskeletal-related protein expression in human osteosarcoma cells. Human 143-B and HOS osteosarcoma cells were treated with various concentrations of timosaponin AIII (TSAIII; 0, 2, 4, and 6 μM) and then harvested to detect the expression and activation of invasive motility-related proteins through immunoblotting. The measured invasive motility-related proteins include (**A**) integrin-αV, integrin-β3, phospho-FAK (focal adhesion kinase) (Y397), total-FAK, phospho-Src, total-Src, (**B**) phospho-cofilin, total-cofilin, testis associated actin remodelling kinase 1 (TESK1), SSH1 (slingshot protein phosphatase 1), phospho-LIM domain kinase 1 and 2 (LIMK1/2), and total-LIMK1/2. Glyceraldehyde-3-phosphate dehydrogenase (GAPDH) was used as the internal control.

Several evidences suggest that integrin/FAK promotes tumor cell migration and invasion through promoting different signaling pathways involving Src family kinases pathway [24,25]. To evaluate the effect of TSAIII on integrin-αv/β3 and FAK/Src kinase expression, human 143-B and HOS cells were treated with various concentrations of TSAIII (0, 2, 4, and 6 μM) through immunoblotting. We found that TSAIII significantly reduced the expression of integrin αV, integrin β3, phosphorylated FAK (Y397) and phosphorylated Src in the 143-B and HOS cells (Figure 3A). Cofilin activity contributes to integrin-mediated cytoskeletal F-actin remodeling and cell migration and invasion by various intracellular and extracellular factors, such as TESK1, LIMKs, and SHH1 [26]. To identify the effect of TSAIII on cofilin activity, TSAIII significantly increased the TESK1 and phosphorylation of cofilin (inactive form) in 143-B and HOS cells; however, the expression of LIMK1/2 and SSH1 was not influenced (Figure 3B).

### 2.4. TSAIII and Cyclo Synergistically Suppress Cell Migration, Invasion, and F-Actin Expression in Human Osteosarcoma Cells

**Figure 4:**
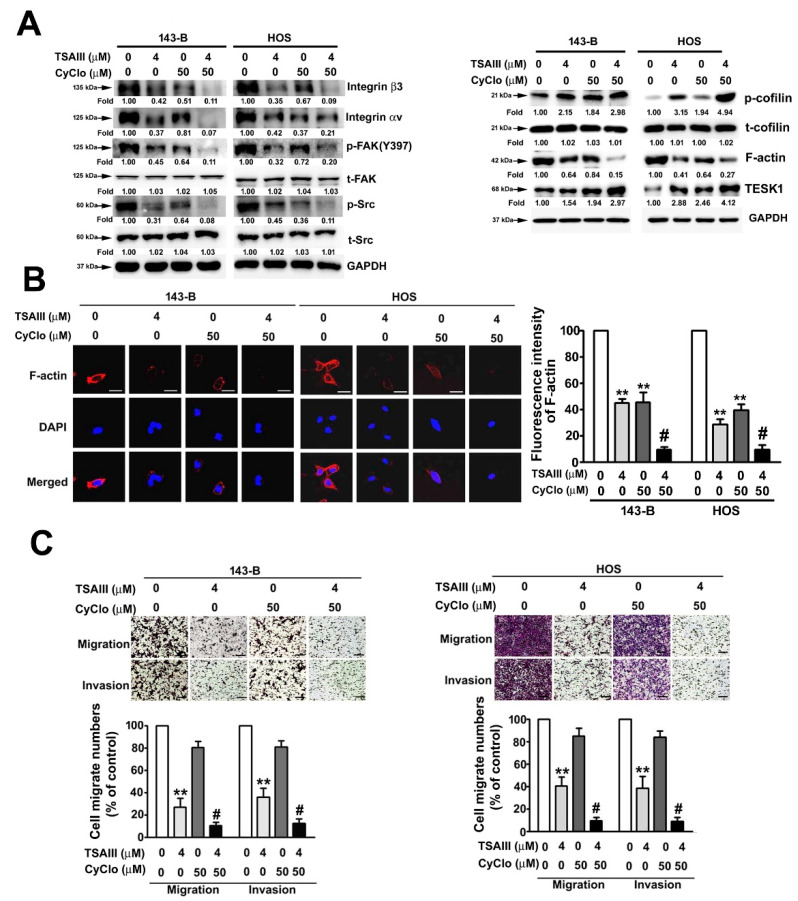
Synergistically inhibitory effect of TSAIII and Cyclo on migration and invasion of human osteosarcoma cells. (**A**) Human 143-B and HOS osteosarcoma cells were treated with various concentrations of TSAIII (0 and 4 μM) and/or Cyclo (0 and 50 μM) and then harvested to detect the expression and activation of cytoskeletal-related proteins through immunoblotting. Glyceraldehyde-3-phosphate dehydrogenase (GAPDH) was used as the internal control. (**B**) A change in the expression of cytoskeletal F-actin was observed in human 143-B and HOS osteosarcoma cells using immunofluorescence analysis. (**C**) The migration and invasion capacities of human 143-B and HOS osteosarcoma cells were measured after treatment with TSAIII in the presence or absence of Cyclo for 18 h (migration) or 24 h (invasion). ** *p* < 0.01 versus control; # *p* < 0.05 versus treatment with Cyclo alone. Cyclo denotes Cyclo(RGDyK) (Intergin inhibitor)**.** Scale bar: 50 μm.

To examine the effect of TSAIII and Cyclo (Cyclo(RGDyK); integrin αVβ3 inhibitor) on the migration and invasion activities of human osteosarcoma cells, human 143-B and HOS cells were treated with TSAIII (0 and 4 μM) and/or Cyclo (0 and 50 μM) and harvested to measure the cytoskeletal-related proteins through immunoblotting. We observed that treatment with TSAIII (4 μM) or Cyclo (50 μM) alone significantly downregulated the expression of integrin-αv, integrin-β3, phosphorylated FAK, phosphorylated Src, and F-actin and upregulated the expression of TESK1 and phosphorylated cofilin (inactive form). Treatment with TSAIII (4 μM) in combination with Cyclo (50 μM) revealed a greater synergistic inhibitory effect on the expression of these cytoskeletal-related proteins (Figure 4A). The effects of TSAIII and Cyclo on F-actin distribution in the 143-B and HOS cells were further assessed using immunofluorescence analysis, and the results confirmed the reduction of F-actin expression by TSAIII and Cyclo (Figure 4B). Treatment with TSAIII (4 μM) alone significantly suppressed cell migration and invasion in human 143-B and HOS cells. Notably, treatment with TSAIII (4 μM) in combination with Cyclo (50 μM) exerted a greater synergistic inhibitory effect on cell migration and invasion in human 143-B and HOS cells (Figure 4C), not affecting the cell viability and cell death in TSAIII combination with Cyclo (50 μM)-treated human 143-B and HOS cells (Figure S1A,C).

### 2.5. TSAIII and PF Synergistically Suppress Cell Migration, Invasion, and F-actin Expression in Human Osteosarcoma Cells

**Figure 5:**
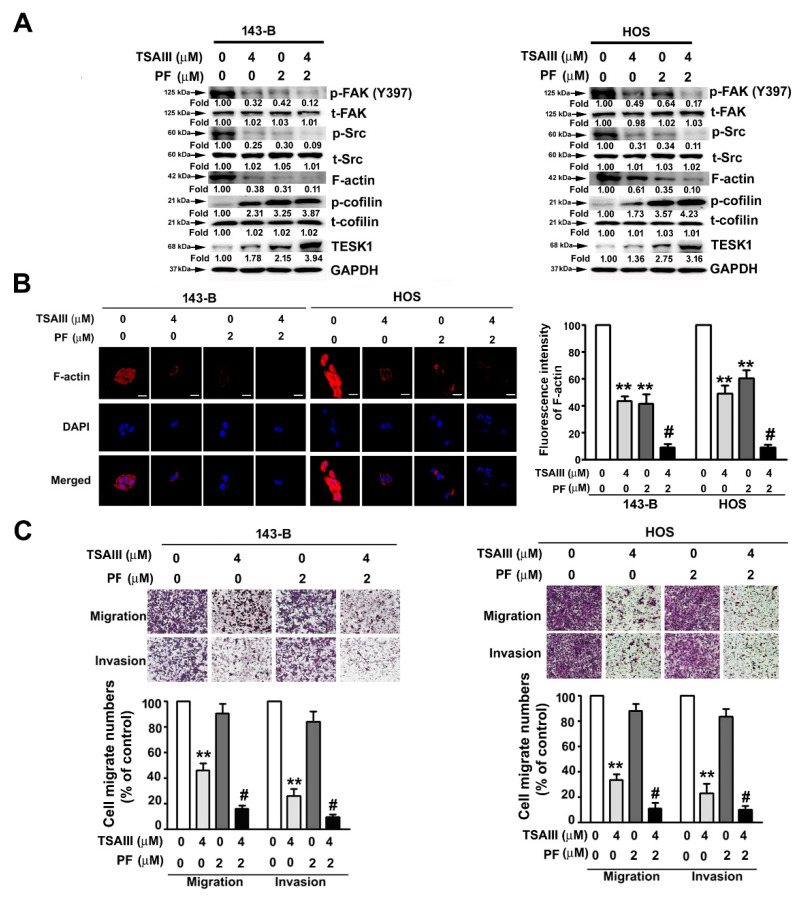
Synergistically inhibitory effect of TSAIII and PF on migration and invasion of human osteosarcoma cells. (**A**) Human 143-B and HOS osteosarcoma cells were treated with various concentrations of TSAIII (0 and 4 μM) and/or PF (0 and 2 μM) and then harvested to detect the expression and activation of cytoskeletal-related proteins through immunoblotting. Glyceraldehyde-3-phosphate dehydrogenase (GAPDH) was used as the internal control. (**B**) Using immunofluorescence analysis, the expression of cytoskeletal F-actin was observed in human 143-B and HOS osteosarcoma cells. (**C**) The migration and invasion abilities of human 143-B and HOS osteosarcoma cells were measured after treatment with TSAIII in the presence or absence of PF for 24 h. ** *p* < 0.01 versus control; # *p* < 0.05 versus treatment with PF alone (mean ± standard error, *n* = 3). PF denotes PF-573228 (focal adhesion kinase inhibitor). Scale bar: 50 μm.

We examined the synergistic effect of TSAIII and PF (PF-573228; FAK kinase inhibitor) on the expression of cytoskeletal-related proteins and the migration and invasion activities in human osteosarcoma cells. As shown in Figure 5A, cotreatment of TSAIII (4 μM) with PF (2 μM) synergistically downregulated the expression of phosphorylated FAK(Y397), phosphorylated Src, and F-actin and upregulated the expression of TESK1 and phosphorylated cofilin (inactive form). The effect of TSAIII and PF on the distribution of F-actin was further observed through immunofluorescence analysis in both cell lines. The results revealed that TSAIII and PF reduced F-actin expression (Figure 5B). Moreover, treatment with TSAIII (4 μM) alone significantly suppressed the cell migration and invasion of human 143-B and HOS cells. However, cotreatment of TSAIII (4 μM) with PF (2 μM) had a significantly synergistic inhibitory effect on the cell migration and invasion abilities of human 143-B and HOS cells (Figure 5C), not affecting the cell viability and cell death in cotreatment of TSAIII (4 μM) with PF (2 μM)-treated human 143-B and HOS cells (Supplementary Figure S1B,D).

### 2.6. TSAII Inhibits Lung Metastasis of Human Osteosarcoma Cells

**Figure 6:**
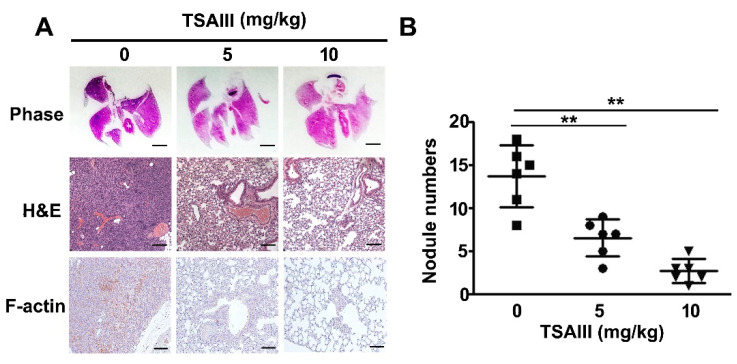
In vivo metastasis of TSAIII in human osteosarcoma cells. For the animal assay of lung metastasis, human osteosarcoma cells were harvested and injected into the tail veins of five-week-old immunodeficient mice (C.B17/IcrPrkdcscid/CrlNarl). The mice were then fed TSAIII (5 and 10 mg/kg) through oral gavage. After two months, the mice were euthanised and (**A**) the histopathology of the lungs in metastatic tumour-bearing animals was analysed. The lungs were fixed in neutral-buffered formalin and stained with haematoxylin and eosin. The F-actin expression were detected with immunohistochemistry assay. (**B**) The nodule numbers were then counted in the mice. ** *p* < 0.01 versus control (*n* = 5). Scale bar: 100 μm.

The tail veins of immunodeficient mice were injected with human osteosarcoma cells, and the mice were treated with TSAIII (0, 5, and 10 mg/kg). Histopathology of lung tissues revealed a notable reduction of tumour mass in the mice treated with TSAIII. Lung metastases of the mice treated with TSAIII were significantly reduced compared with the control group (Figure 6A). We also found that TSAIII markedly reduced the expression of F-actin by immunohistochemistry assay (Figure 6A). A marked reduction in the number of lung nodules was observed in the mice treated with 5 or 10 mg/kg of TSAIII (Figure 6B). These results indicated that TSAIII significantly inhibits human osteosarcoma cell-mediated lung metastasis in vivo.

## 3. Discussion

**Figure 7:**
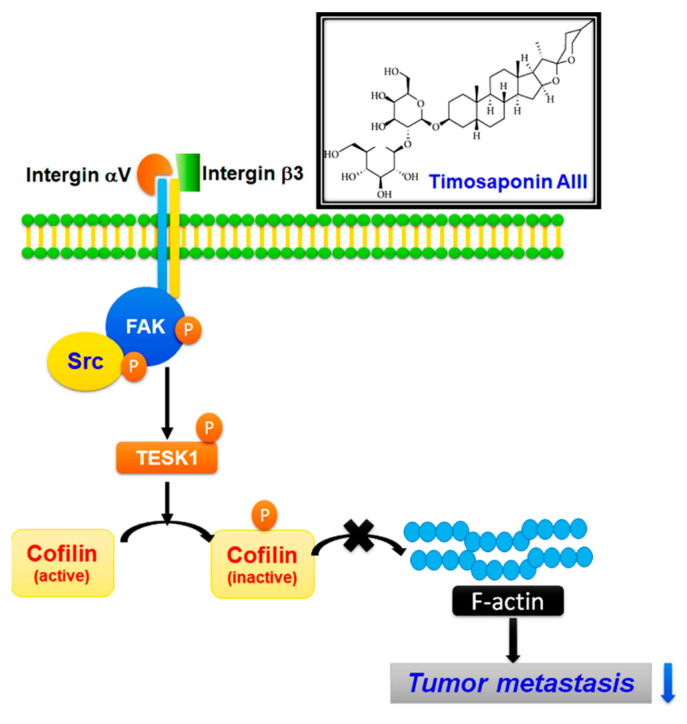
TSAIII suppresses metastasis of human osteosarcoma cells and underlying molecular mechanism.

Despite the development of diverse anticancer agents for osteosarcoma treatment, the survival rate of patients is still low. Thus, exploring novel and potential treatment strategies and developing new anticancer drugs for treating osteosarcoma and preventing recurrence and metastasis are urgently required. Exploring the potential of natural phytochemicals is noteworthy because plant-derived compounds have been shown to possess potential bioactivities against cancer progression. TSAIII is one of the major compounds extracted from *A. asphodeloides*, which is used as a traditional herb. The present study results are as follows: (I) TSAIII did not decrease the cell growth and colony formation in human osteosarcoma cells. (II) TSAIII significantly inhibited cell migration and invasion by downregulating the expression and distribution of cytoskeletal F-actin. (III) TSAIII significantly inhibited migration and invasion through the downregulation of integrin-αVβ3, phosphorylated FAK(Y397), and phosphorylated Src and the upregulation of TESK1 and phosphorylated cofilin. (IV) Cotreatment models of TSAIII plus Cyclo or PF more synergistically reduced the expression and activation of these cytoskeletal-related proteins and disruption of F-actin cytoskeleton, consequently blocking the migration and invasion capacities of human osteosarcoma cells. (V) TSAIII significantly inhibited in vivo osteosarcoma metastasis mouse model. These findings indicate that TSAIII treatment is a novel strategy for the auxiliary treatment of osteosarcoma (Figure 7).

Pulmonary metastasis is the major cause of morbidity and mortality. Cytoskeleton actin is a highly dynamic structure that is responsible for cell motility, adhesion, and growth during cancer progression [27]. Cofilin plays an essential role during tumorigenesis and metastasis by regulating F-actin dynamics [28] and cofilin activity is spatially and temporally regulated by various intracellular molecules and mechanisms. The activation of cofilin is regulated through phosphorylation by cofilin-binding molecules. Phosphorylation at Ser-3 by LIMKs and TESKs leads to the deactivation of cofilin; by contrast, dephosphorylation by SSHs and PDXP leads to the reactivation of cofilin [16,29]. TESKs are related to LIMKs and contain the highly homologues kinase domain of LIMKs at the N-terminus and a unique C-terminal proline-rich domain. TESK1 acts downstream of integrins and plays a critical role in integrin-mediated actin reorganization by phosphorylating and deactivating cofilin [30]. High TESK1 protein expression is characterized in Wilms’ tumours (WTs) and could be essential for WT onset [31]. The activation of transcriptional coactivators YAP and TAZ through tight coupling to the actin cytoskeleton architecture has been shown to promote resistance to anticancer therapies. The actin remodelling dynamics of TESK1 confers BRAF inhibitor (PLX4032) resistance to melanoma cells through YAP/TAZ activation [12]. In the present study, TSAIII significantly increased the expression of TESK1 and deactivated cofilin through phosphorylation at serine 3. Both cotreatment model TSAIII plus Cyclo(RGDyK) and TSAIII plus PF exerted greater synergistic inhibitory effects on cofilin activation and F-actin expression by increasing TESK1 expression, independent of SSH expression, thereby leading to the inhibitory effect on cell migration and invasion.

Integrins contribute to cancer angiogenesis and metastasis by triggering distinct intracellular signalling and regulating the reorganisation of actin filaments in response to microenvironmental changes [7,8,32]. Periodontal pathogens promote the highly aggressive phenotype of oral cancer through crosstalk between integrin/FAK and TLR/MyD88 signaling [33]. Integrins αV and β3 mediate TGFβ1 (a transforming growth factor β1-induced extracellular matrix protein)-induced endogenous anti-tumour and antiangiogenic properties [34]. The regulation of dynamic actin polymerization by cofilin serine 3 phosphorylation is dependent on the activity of integrin-linked kinase. The c-Src complex during cell adhesion [35]. In the present study, TSAIII significantly inhibited the metastasis of human osteosarcoma cells by deactivating cofilin through the integrin αVβ3/FAK/Src signalling pathway.

Plant-derived compounds have bioactive potential and are explored as an auxiliary agent for anticancer therapy and for improving the outcomes of patients with cancer. Autophagy inhibition enhances the TSAIII-induced lung cancer cell apoptosis and antitumour effect in vitro and in vivo [36]. TSAIII exerts significant anti-tumour effects on Taxol-resistant human lung cancer cells and ovarian carcinoma cells [37]. TSAIII show the antitumor effect on inducing cell apoptosis and autophagy in T cell acute lymphoblastic leukemia (T ALL) Jurkat cells through the inhibition of PI3K/Akt/mTOR pathway [38]. TSAIII can induce apoptosis and inhibit cell proliferation by suppressing the STAT3 and ERK1/2 pathways in human pancreatic cancer [39]. Moreover, TSAIII demonstrates significant antimetastatic activity against renal cell carcinoma cells through the inhibition of cathepsin C expression at the AKT/miR-129-5p axis [40]. In the present study, TSAIII significantly inhibited cell migration and invasion of human osteosarcoma cells in vitro. An analysis of the molecular machinery suggested that TSAIII suppressed the invasive motility through downregulation of integrin-αv and integrin-β3, phosphorylation of FAK/Src, and activation of TESK1 and cofilin expression, thus leading to the destruction of the F-actin cytoskeleton.

## 4. Materials and Methods

### 4.1. Chemical Reagents and Antibodies

TSAIII (CFN98151) was obtained from ChemFaces (Wuhan, Hubei, PRC). The CCK8 kit was obtained from Sigma-Aldrich (St. Louis, MO, USA). Primary antibodies against F-actin (NB100-64792SS) were purchased from Novus Biologicals (Centennial, CO, USA). Integrin αV (4711S), integrin-β3 (4702S), phospho-Tyr397-FAK (3283S), total-FAK (3285S), phospho-Y416-Src (2101S), total-Src (2123S), phospho-Ser3-cofilin (3313S), total-cofilin (5175T), TESK1 (4655T), SSH1 (13578T), phospho-LIMK1-Thr508/LIMK2-Thr505 (3841S), total-LIMK1/2 (3845T), and glyceraldehyde-3-phosphate dehydrogenase (GAPDH, 2118S) were purchased from Cell Signaling Technology (Danvers, MA, USA). PF-573228 (FAK kinase inhibitor) and Cyclo(RGDyK) (integrin αVβ3 inhibitor) were purchased from MedChem Express (NJ, USA).

### 4.2. Cell Culture

Cell culture was performed as described in a previous study [41]. Human 143-B and HOS osteosarcoma cell lines were kindly provided by Prof. Shun-Fa Yang (Institute of Medicine, Chung Shan Medical University, Taichung, Taiwan). The normal mouse osteoblast cell line MC3T3-E1 was kindly provided by Prof. Chih-Hsin Tang (Department of Pharmacology, China Medical University, Taichung, Taiwan). The 143-B, HOS, and MC3T3-E1 cells were cultured in MEM (HyClone, UT, USA) containing 10% Fetal bovine serum (FBS) and 100 U/mL penicillin–streptomycin (Invitrogen Life Technologies, Carlsbad, CA, USA). All cell cultures were maintained in a humidified incubator under 5% CO_2_ at 37 °C.

### 4.3. Cell Viability Assay

To determine the cytotoxicity of TSAIII in 143-B, HOS, and MC3T3-E1 cells, the cells (8 × 10^3^/100 μL) were seeded in 24-well plates and treated with various concentrations (0, 2, 4, 6, 8, 10 μM) of TSAIII for 24 and 48 h. The cells were then cultured with fresh medium containing the CCK8 solution (100 μL/well) for 2 h. Cell viability was measured at 405 nm by using a Multiskan MS ELISA reader (Labsystems, Helsinki, Finland).

### 4.4. Colony Formation Assay

Human 143-B and HOS osteosarcoma cells were seeded in six-well plates (1 × 10^4^/well) and treated with various concentrations of TSAIII for 7 days. More than 100 colonies were stained with 0.5% crystal violet solution for 30 min at room temperature and observed. Three independent experiments were performed.

### 4.5. Annexin V/PI Staining by Flow Cytometry Analysis

An apoptosis assay was performed as previously described [42]. Human osteosarcoma cells (5 × 10^5^/well) were treated with various concentrations of TSAIII for 24 h and then fixed with 75% ice ethanol overnight. The fixed cells were then stained with Propidium Iodide (PI) reagent for 20 min. Cell DNA content was measured through flow cytometry by using the Muse Cell Analyzer (Merck Millipore, Burlington, MA, USA). Outcome data further was analysed using the Muse Cell Analyzer. An apoptosis assay was performed as previously described [41]. After the human osteosarcoma cells were treated with various concentrations of TSAIII for 24 h, cells were harvested and apoptosis was measured using the Muse Annexin V and Dead Cell Assay Kit (Merck Millipore). Briefly, the collected cells were incubated with 5 μL of Annexin V-FITC and 5 μL of PI reagents at room temperature in darkness for 15 min. The apoptotic cell population was then analysed using the Muse Cell Analyzer (Merck Millipore).

### 4.6. Immunoblotting Analysis

Immunoblotting analysis was performed as previously described [42]. Briefly, proteins were harvested from human osteosarcoma cells and lysed with lysis buffer. Equal amounts of total protein (20 μg) from each experimental group were subjected to 10%–12% SDS-PAGE for protein separation and then transferred onto a PVDF membrane (Life Technologies, Carlsbad, CA, USA). The membranes were blocked with 5% nonfat dry milk in Tris-buffered saline with Tween-20 buffer. The blocked membranes were further incubated with target primary antibodies and subsequently with secondary antibodies to detect antibody-bound protein bands by using the Luminescent Image Analyzer (LAS 4000 mini, GE Healthcare Bio-Sciences, Pittsburgh, PA, USA).

### 4.7. Migration and Invasion Assay

In vitro cellular migration and invasion were analyzed as previously described [42]. Briefly, human osteosarcoma cells (5 × 10^5^/well) were seeded onto filter inserts (pore size, 8 μm) precoated with or without Matrigel (0.5 mg/mL) for cellular invasion assay and migration assay, respectively. Osteosarcoma cells that migrated to or invaded the lower side of the filter insert were stained with 5% Giemsa reagent and counted at 200× magnification. Four microscopic fields were counted for each filter, and each experiment was repeated three times.

### 4.8. In Vivo Metastasis Animal Experiments and Immunohistochemistry

Lung metastasis assay was performed as previously described [43]. The protocol was approved by the Institutional Animal Care and Use Committee (approval number 2196) and conformed to the institutional animal welfare guidelines of Chung Shan Medical University. Immunodeficient nude mice (C.B17/IcrPrkdcscid/CrlNarl) were obtained from the National Laboratory Animal Center. Human 143-B osteosarcoma cells (1 × 10^6^ cells) suspended in 0.1 mL of PBS were injected into the tail vein of mice. Mice were randomly divided into three groups (*n* = 5 per group) and fed TSAIII (5 and 10 mg/kg of body weight, daily) through oral gavage. After 2 months, the mice were euthanised with CO_2_. Subsequently, the lungs were isolated and fixed in 5% neutral-buffered formalin. Tissue sections were collected and stained with H and E (haematoxylin and eosin) for morphological analysis and immunohistochemistry staining for F-actin expression.

### 4.9. Statistical Analysis

Each experiment was repeated at least three times. Results are presented as the mean ± standard error. One-way analysis of variance (ANOVA) followed by Dunnett post hoc test and statistical comparisons were made using Student’s t test and SPSS (version 18.0). Significance was defined at the *p* < 05 or 01 levels.

## 5. Conclusions

TSAIII demonstrated antimetastasis activity in vivo and in vivo in human osteosarcoma cells. These results provide antimetastatic effect and molecular mechanism for TSAIII against human osteosarcoma.

## Data Availability

The authors will freely release all data underlying the published paper upon direct request to the corresponding author.

## Supplementary Materials

The following are available online at https://www.mdpi.com/1424-8247/14/3/260/s1, Figure S1: Synergistically inhibitory effect of TSAIII and Cyclo or PF on cell viability and cell death of human osteosarcoma cells. (A, B) 143-B and HOS cells were treated to TSAIII (0 and 4 μM) and/or Cyclo (0 and 50 μM) and PF (0 and 2 μM) for 24 h, and cell viability was measured using the CCK8 assay. (C.D) Cell death in human 143-B and HOS osteosarcoma cells were detected using Annexin V/PI staining by flow cytometry. Cyclo denotes Cyclo(RGDyK); PF denotes PF-573228.
