# Supplementary material for: Potential Antimetastatic Effect of Timosaponin AIII against Human Osteosarcoma Cells through Regulating the Integrin/FAK/Cofilin Axis
Source: Pharmaceuticals (Basel). 2021 Mar 14;14(3):260. doi: 10.3390/ph14030260 (PMC8000016; doi:10.3390/ph14030260)
Supplement: Supplementary file 1 [file pharmaceuticals-14-00260-s001.pdf]

Potential antimetastatic effect of Timosaponin AIII against human osteosarcoma cells through regulating the Integrin/FAK/cofilin axis

Yi-Hsien Hsieh [1,2,†], Wen-Hung Hsu [3,†], Shun-Fa Yang [1,2], Chung-Jung Liu [3], Ko-Hsiu Lu [4,5], Pei-Han Wang [1] and Renn-Chia Lin [4,5,*]

Supplementary Figure 1

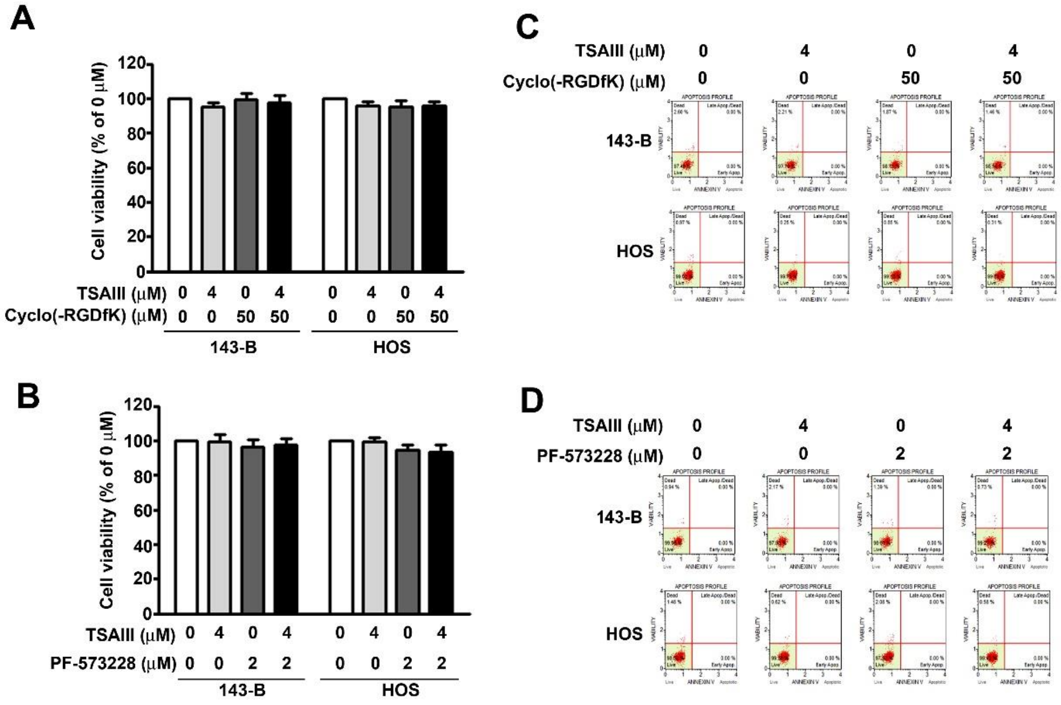

Figure S1: Synergistically inhibitory effect of TSAIII and Cyclo or PF on cell viability and cell death of human osteosarcoma cells. (A, B) 143-B and HOS cells were treated to TSAIII (0 and 4 μM) and/or Cyclo (0 and 50 μM) and PF (0 and 2 μM) for 24 h, and cell viability was measured using the CCK8 assay. (C.D) Cell death in human 143-B and HOS osteosarcoma cells were detected using Annexin V/PI staining by flow cytometry. Cyclo denotes Cyclo(RGDyK); PF denotes PF-573228.

.
